# Sleep Disturbances in Frontline Health Care Workers During the COVID-19 Pandemic: Social Media Survey Study

**DOI:** 10.2196/27331

**Published:** 2021-05-19

**Authors:** Nancy H Stewart, Anya Koza, Serena Dhaon, Christiana Shoushtari, Maylyn Martinez, Vineet M Arora

**Affiliations:** 1 Department of Medicine University of Kansas Medical Center Kansas City, KS United States; 2 Kansas City University Kansas City, MO United States; 3 University of Illinois Chicago, IL United States; 4 Department of Internal Medicine Advocate Lutheran General Hospital Park Ridge, IL United States; 5 Department of Medicine University of Chicago Medical Center Chicago, IL United States

**Keywords:** social media, sleep disorders, frontline health care workers, burnout, insomnia, sleep, health care worker, stress, survey, demographic, outcome, COVID-19

## Abstract

**Background:**

During the COVID-19 pandemic, health care workers are sharing their challenges, including sleep disturbances, on social media; however, no study has evaluated sleep in predominantly US frontline health care workers during the COVID-19 pandemic.

**Objective:**

The aim of this study was to assess sleep among a sample of predominantly US frontline health care workers during the COVID-19 pandemic using validated measures through a survey distributed on social media.

**Methods:**

A self-selection survey was distributed on Facebook, Twitter, and Instagram for 16 days (August 31 to September 15, 2020), targeting health care workers who were clinically active during the COVID-19 pandemic. Study participants completed the Pittsburgh Sleep Quality Index (PSQI) and Insomnia Severity Index (ISI), and they reported their demographic and career information. Poor sleep quality was defined as a PSQI score ≥5. Moderate-to-severe insomnia was defined as an ISI score >14. The Mini-Z Burnout Survey was used to measure burnout. Multivariate logistic regression tested associations between demographics, career characteristics, and sleep outcomes.

**Results:**

A total of 963 surveys were completed. Participants were predominantly White (894/963, 92.8%), female (707/963, 73.4%), aged 30-49 years (692/963, 71.9%), and physicians (620/963, 64.4%). Mean sleep duration was 6.1 hours (SD 1.2). Nearly 96% (920/963, 95.5%) of participants reported poor sleep (PSQI). One-third (288/963, 30%) reported moderate or severe insomnia. Many participants (554/910, 60.9%) experienced sleep disruptions due to device use or had nightmares at least once per week (420/929, 45.2%). Over 50% (525/932, 56.3%) reported burnout. In multivariable logistic regressions, nonphysician (odds ratio [OR] 2.4, 95% CI 1.7-3.4), caring for patients with COVID-19 (OR 1.8, 95% CI 1.2-2.8), Hispanic ethnicity (OR 2.2, 95% CI 1.4-3.5), female sex (OR 1.6, 95% CI 1.1-2.4), and having a sleep disorder (OR 4.3, 95% CI 2.7-6.9) were associated with increased odds of insomnia. In open-ended comments (n=310), poor sleep was mapped to four categories: children and family, work demands, personal health, and pandemic-related sleep disturbances.

**Conclusions:**

During the COVID-19 pandemic, nearly all the frontline health care workers surveyed on social media reported poor sleep, over one-third reported insomnia, and over half reported burnout. Many also reported sleep disruptions due to device use and nightmares. Sleep interventions for frontline health care workers are urgently needed.

## Introduction

Since March 2020, the pandemic of SARS-CoV-2, the virus that causes COVID-19, has impacted people’s lives on a global scale, especially the lives of health care workers on the front lines. During the pandemic, health care workers have used social media to share their experiences serving on the front lines, such as lack of personal protective equipment, mental anguish, burnout [1], and the toll of caring for patients with COVID-19. Repeatedly, health care workers have reported concerns that include sleep loss and other sleep disturbances [2]. Although these sleep issues certainly can be attributed to long working hours, they may also occur for other reasons. Social media posts suggest that health care workers are suffering from nightmares, insomnia, and using mobile devices at night to review social media (“doomscrolling”) [3,4]. Given the increased professional and personal responsibilities of health care workers during the pandemic, it is possible that sleep loss or disturbances will be compounded by additional worries during the pandemic. Moreover, mounting evidence is demonstrating that inadequate physician sleep is associated with burnout and medical errors [5,6].

Several studies in various countries have described the sleep of health care workers during the COVID-19 pandemic. Most notable are two meta-analyses on sleep in health care workers during the initial months of the COVID-19 pandemic, with studies mostly focusing on the experience of health care workers in China. Pappa et al [7] reviewed 5 cross-sectional studies conducted among Chinese health care workers prior to April 17, 2020, and reported a prevalence of insomnia (38.9%) among them. Salari et al [8] reviewed 7 cross-sectional studies from Asia and the Middle East conducted among nurses and physicians prior to June 24, 2020, and they reported a prevalence of sleep disturbances of 34.8% and 41.6% in nurses and physicians, respectively. Additionally, several other studies reported on sleep loss in health care workers from countries such as Oman [9], Bahrain [10], and Spain [11]. 

Prior to the COVID-19 pandemic, studies reported that nearly 1 in 2 physicians admitted experiencing symptoms of burnout [12]. In 2004, researchers described the profound impact of perceived sleep loss on trainee well-being [13]. That same year, Lockley et al [14] demonstrated that reduction of intern work hours improved sleep and decreased attention failures. In 2015, the American Thoracic Society published an official statement affirming that good quality and quantity of sleep is imperative for healthy physicians [15].

To date, few studies of the sleep experience among frontline health care workers during the COVID-19 pandemic in the United States have been conducted. These studies did not utilize validated measures of sleep and were limited to a single institution [3,16]. In the United States, to our knowledge, no study has yet evaluated sleep using standardized measures in clinically active frontline health care workers during the COVID-19 pandemic. Despite anecdotal observations of sleep loss described via social media platforms, no study has yet explicitly explored the sleep of frontline health care workers during the COVID-19 pandemic utilizing social media, an increasingly important and influential mode of communication during the pandemic. The aim of this study was to describe the sleep disturbances of a sample of predominantly US frontline health care workers during the COVID-19 pandemic with validated instruments through a survey distributed on social media. A secondary aim of this study was to examine the association between the demographic and career characteristics of health care workers and their reported sleep disturbances and burnout.

## Methods

From August 31 to September 15, 2020, we recruited frontline health care workers from across the world to complete the web-based R.E.S.T. (Recommending Essential Sleep Time) survey. The restricted, self-selection survey (respondents chose to participate by clicking on the survey link) was distributed through Twitter, Facebook, and Instagram social media accounts created specifically for the study. A river sampling method was used to recruit participants anonymously [17]. This web-based sampling technique redirected interested participants to the survey link via digital promotion (eg, banners, advertisements, flyers, offers) [17]. A unique bit.ly link was created for the social media accounts to enable tracking of views and clicks. Participation in the study was voluntary, and participants could opt out at any time while responding to the survey questions. Participants included physicians, physician assistants, nurse practitioners, nurses, pharmacists, physical/ occupational/ respiratory therapists, health care trainees, and allied health care workers (eg, medical technologists, therapists, certified nursing assistants). The University of Chicago Institutional Review Board reviewed and approved the protocol.

### Survey Design

Study data were collected and managed using Research Electronic Data Capture (REDCap), hosted at the University of Chicago. REDCap is a secure, web-based software platform that supports data capture for research studies [18,19]. Qualifying participants for our study were at least 18 years of age and were frontline health care workers who were clinically active during the COVID-19 pandemic. The survey automatically terminated for respondents who did not meet these inclusion criteria. We asked the participants to frame all their responses for 1 month in the pandemic in which they experienced the greatest clinical intensity and risk of COVID-19 transmission. This time frame was selected as the most representative of the sleep of respondents while they were clinically active on the front lines caring for patients with COVID-19.

The survey contained the following categories: Sleep, Well-being, Career, and Baseline Demographics. The Sleep section included the Pittsburgh Sleep Quality Index (PSQI) [20] and the Insomnia Severity Index (ISI) [21]. The PSQI is a 19-item validated questionnaire that measures sleep quality over 1 month, generating 1 global score [20]. The ISI is a 7-item validated questionnaire that assesses the nature, severity, and impact of insomnia in adults [21]. The Sleep section also included questions about sleep disturbances due to device use. The Well-being section included the Mini-Z Burnout Survey, an 8-item validated questionnaire that investigates health care burnout and job satisfaction [22]. The Mini-Z also includes a 1-item burnout measure that is validated against the longer Maslach Burnout Inventory [23]. Other questions in this section related to pandemic-related worries, changing responsibilities, living situation, and health status. The Career section collected data on each participant’s job role, level of training, specialty, and career setting prior to the pandemic and whether they cared for patients with COVID-19 directly. Baseline demographics were collected on marital status, age, gender, race, ethnicity, type of practice, location of practice, and type of community served (eg, urban, suburban, rural). Two open-ended questions were also included in the survey: “What was the cause of your sleep disruption(s) during your reported month?” and “Is there anything else you would like to share?”

Upon completion of the survey, participants were invited to submit their email address to enter a daily raffle for a US $25 Amazon gift card (14 gift cards in total). Before deployment, the survey was piloted with a few health care workers who were not affiliated with the study. Changes made included deleting duplicate questions and removing one sleep scale to abbreviate the time to complete the survey to 10 minutes or less.

### Social Media Distribution Strategy

To disseminate the survey link, we created a Facebook page and a Twitter and Instagram account on behalf of the R.E.S.T. study. This enabled the use of engagement tracking built into these social media platforms to calculate the response rate. To market our survey through Instagram, we used paid advertisements. On Facebook, we posted on health care–specific social media groups (eg, Women Physician Wellness, StyleMD, and the Physician Collective). On Twitter, we tagged colleagues and organizations and employed health care–specific hashtags (eg, #MedTwitter, #NurseTwitter). Although the authors re-shared the R.E.S.T. survey posts on their personal Twitter accounts to increase visibility, the link remained connected to the original posts of the R.E.S.T. accounts to preserve the ability to track engagement. To promote the survey, an infographic was posted each day on the study’s social media accounts. Survey links were also placed in the biography of each social media account. To draw attention to the survey post and the account, we linked each post to standard information from national organizations (eg, the American Academy of Sleep Medicine and the Sleep Research Society) and to articles about sleep and the pandemic through the study’s social media accounts.

One of our study authors (SRD) used a customized *bit.ly* link [24] to track user engagement daily on Facebook, Twitter, Instagram, and email. The metrics analytics from the social media platforms enabled us to view specific metrics on reach (the total number of individuals who viewed the content) and link clicks (the number of times individuals chose to open the survey). At midnight on each day, we calculated the reach and link clicks of the study on each social media website. This enabled us to compare the performance of each platform and track our progress. We also calculated the number of survey responses per day while the survey was active.

### Statistical Analysis

Descriptive statistics were used to quantify the participants’ demographics and profession. Primary outcomes were poor sleep quality and moderate to severe insomnia. Poor sleep quality was defined as a PSQI score ≥5 [20]. Moderate-to-severe insomnia was defined as an ISI score of >14.21. The Mini-Z Burnout Survey was used to measure burnout [22]. Multivariate logistic regression was used to test for independent associations between age, gender, race (Black vs non-Black), ethnicity (Hispanic vs non-Hispanic), and profession (physician vs. nonphysician), with odds of each of the outcomes, after controlling for those with a preexisting sleep disorder. All statistical analyses were conducted using Stata Statistical Software, Release 16 (StataCorp LLC), with *P*<.05 used to indicate statistical significance.

### Coding Open-ended Comments

There were two open-ended questions in the survey. For participants who responded to one or both questions, open coding of comments was used [25]. Sleep disturbance was mapped to four main themes: (1) children and family; (2) work demands affecting sleep; (3) personal health conditions; and (4) pandemic-related sleep disturbances. Subthemes were created for each of these themes. 

## Results

Our social media posts were seen by 87,061 unique individuals and resulted in 976 clicks on our survey link. Our final sample contained 963 submitted surveys. The most common ways people discovered the survey were through Twitter (372/944, 39.4%), in a private Facebook group (199/944, 21.1%), and through a colleague (103/944, 10.9%). 

Participants were mostly female (707/963, 73.4%), White (894/963, 92.8%), aged 30-49 years (692/963, 71.9%), and physicians (620/963, 64.4%) (Table 1).

**Table 1:** Characteristics of the sample of survey respondents (N=963).

Characteristic		Value, n (%)
**Age group (years)**		
	18-29	149 (15.5)
	30-49	692 (71.9)
	50+	86 (8.9)
	Did not answer	36 (3.7)
**Gender**		
	Male	220 (22.9)
	Female	707 (73.4)
	Did not answer	36 (3.7)
**Race^a^**		
	White	894 (92.8)
	Black	69 (7.2)
**Ethnicity^b^**		
	Non-Hispanic	759 (78.8)
	Hispanic	124 (12.9)
	Did not answer	80 (8.3)
**Significant other**		
	Yes	700 (72.7)
	No	220 (22.9)
	Did not answer	43 (4.5)
**Career role**		
	Physician	620 (64.4)
	Nonphysician	343 (35.6)
	Directly cared for patients with COVID-19	744 (80)
**Country or continent of residence**		
	United States	882 (91.6)
	Canada	18 (1.9)
	Central America	10 (1.0)
	South America	9 (0.9)
	Europe	7 (0.7)
	Asia/Oceania	4 (0.4)
	Australia/New Zealand	4 (0.4)
	Africa	3 (0.3)
	Did not answer	26 (2.7)

^a^Reference group for race: non-Black.

^b^Reference group for ethnicity: non-Hispanic.

Poor sleep quality, defined as a PSQI score ≥5, was identified in 95.5% (920/963) of all health care workers (Table 2). Rates of moderate and severe insomnia were 249/963 (28.5%) and 39/963 (4.5%), respectively (Table 2). Average sleep duration was 6.1 hours per day (SD 1.2) (Table 2).

Many respondents (554/910, 60.9%) reported sleep disruptions due to device use or due to nightmares at least once per week (420/929, 45%). Those with a sleep disorder were more likely to report burnout (52.1% vs 63.2%, χ^2^_4_=116.4; *P*<.001). In unadjusted analyses, being Black (31.0% vs 59.4%), having Hispanic ethnicity (27.9% vs 59.6%), being single (44.6% vs 27.1%), and being a non-physician (50.7% vs 23.2%) were significantly associated with risk of moderate-to-severe insomnia. Non-Hispanic ethnicity was the reference group. In the multivariable logistic regressions, not being a physician (odds ratio [OR] 2.4, 95% CI: 1.7-3.4), caring for patients with COVID-19 (OR 1.8, 95% CI 1.2-2.8), Hispanic ethnicity (OR 2.2, 95% CI 1.4-3.5), and being female (OR 1.6, 95% CI 1.1-2.4) were associated with increased odds of insomnia (Table 3). Having a sleeping disorder also increased the odds of insomnia compared to those without one (OR 4.3, 95% CI 2.7-6.9) (Table 3). With respect to confounders, we did not focus on low socioeconomic status; however, we did evaluate geography, and we noted no major impact on geography.

**Table 2:** Prevalence of poor sleep and insomnia in health care workers working during the COVID-19 pandemic (N=963).

Variable		Value
Poor sleep quality^a^, n (%)		920 (95.5)
**Insomnia^b^** **, n (%)**		
	Absent	220 (25.1)
	Subthreshold	367 (41.9)
	Moderate	249 (28.5)
	Severe	39 (4.5)
Sleep duration (hours)^c^, average (SD)		6.1 (1.2)

^a^As measured by the Pittsburgh Sleep Quality Index (PSQI); poor sleep quality was defined as a PSQI score ≥5 [20].

^b^As measured by the Insomnia Severity Index (ISI); insomnia was qualified by ISI score, interpreted as absent (0-7), subthreshold (8-14), moderate (15-21), or severe (22-28) [21].

^c^Average hours of sleep duration was defined by the PSQI [20].

**Table 3:** Risk factors for insomnia among frontline health care workers during the COVID-19 pandemic (N=963).

Characteristic		Insomnia^a^, odds ratio (95% CI)	*P* value
**Age group (years)**			
	18-29	Reference	N/A^b^
	30-49	1.1 (0.7-1.8)	.63
	≥50	1.3 (0.7-2.4)	.49
**Gender**			
	Male	Reference	N/A
	Female	1.6 (1.1-2.4)	.02
Black race		1.5 (0.8-2.7)	.24
Hispanic ethnicity		2.2 (1.4-3.5)	<.001
No significant other		1.3 (0.9-1.9)	.18
**Career role**			
	Physician	Reference	N/A
	Nonphysician	2.4 (1.7-3.4)	<.001
Prior sleep disorder		4.3 (2.7-6.9)	<.001
Cared for patients with COVID-19		1.8 (1.2-2.8)	.008

^a^As measured by the Insomnia Sleep Index (ISI); insomnia was defined by ISI scores qualifying as moderate to severe insomnia (>14) [21].

^b^N/A: not applicable.

A total of 310 open-ended comments regarding factors affecting sleep were categorized into the following four themes: (1) children and family; (2) work demands affecting sleep; (3) personal health conditions; and (4) pandemic-related sleep disturbances. The most frequently reported non–pandemic-related theme was children and family (n=59) (“COVID plus home stress plus stress over my kids, my job, my marriage”) (Table 4). Other non–pandemic-related sleep disturbances included work demands affecting sleep (n=48) (“The volume of calls and messages from my patient and caregiver population is through the roof and I’m sleeping 4-5 hours per night”) and personal health (n=41) (“Insomnia predating COVID, but worsened with COVID”) (Table 4). Pandemic-related sleep disturbances (n=48) (“I never had sleep issues prior to the COVID-19 pandemic; suddenly I had issues with sleep initiation”) were also noted (Table 4).

**Table 4:** Themes, subthemes, and example quotes from the open-ended comments (n=310).

Theme		Subthemes	n (%)	Example quote
**Sleep disruptions not due to the pandemic**				
	Children and family (n=59)	Child care (1-18 years)	27 (46)	“6 year old who wakes 2-3 times per night (some secondary to COVID related anxiety for him), 2.5 year old who wakes about 2-3 nights per week”
		Infant care	18 (31)	“Both COVID and my usual sleep disturbances plus my infant daughter”
		Concerns about family affecting sleep	8 (17)	“COVID plus home stress plus stress over my kids, my job, my marriage”
		Pregnancy affecting sleep	6 (10)	“I was pregnant with twins and carried to term. COVID increased my anxiety and work-related stress. I had to lay off employees and reduce my salary, right before maternity leave.”
	Work demands affecting sleep (n=48)	Shift work	18 (38)	“Mostly schedule fluctuations each week - late evening shifts followed by early morning shifts”
		Work-life interference	18 (38)	“Extra work at home [charting] due to increased patient volumes”
		Residency training	6 (13)	“Switching schedules with residency (days/nights and 28-hour calls), overall sleep debt, and fatigue”
		Volume of calls/pages	6 (13)	“The volume of calls and messages from my patient and caregiver population is through the roof…sleeping 4-5 hours [per night].”
	Personal health conditions (n=41)	Poor sleep prior to the pandemic	18 (44)	“I have never been a great sleeper.”
		Chronic medical issue	12 (29)	“Possibly COVID-19 related.... I had an onset of Obsessive-Compulsive Disorder this summer and have been adjusting to antipsychotic medication, intervention, and prescription sleep aids.”
		Formal sleep disorder	8 (20)	“Insomnia pre-dating COVID, but [it] worsened with COVID”
		Female hormones	3 (7)	“In vitro fertilization side effects”
**Sleep disruptions due to the pandemic **				
	Pandemic-related sleep disturbances (n=48)	Pandemic affecting sleep	17 (35)	“I never had sleep issues prior to the COVID-19 pandemic. Suddenly I had issues with sleep initiation.”
		Nightmares about the pandemic	8 (17)	“The only thing that has changed with my sleep is an increase in nightmares and night terrors primarily regarding work situations.”
		Pandemic-related exhaustion	8 (17)	“In the last 1-2 months, I am EXHAUSTED and fall asleep easily, often on the couch right after dinner.”
		Medication/substance use to cope with the pandemic	7 (15)	“Alcohol plays a role in my sleep deprivation and ‘coping’ during COVID.”
		Worry about pandemic response	4 (8)	“I worry about how COVID is being managed by the President of the United States and I don't believe he is doing a good job; I worry things will get even worse if he is elected to a second term. This does keep me awake at night.”

## Discussion

### Principal Findings

To our knowledge, this is the first study to evaluate self-reported sleep disturbances among a sample of predominantly US frontline health care workers during the COVID-19 pandemic with validated instruments using a survey distributed on social media. Our study demonstrates that nearly all (96%) of the surveyed health care workers reported poor sleep, over 30% reported moderate to severe insomnia, and over 50% reported burnout. A majority of respondents also reported sleep disruptions due to personal technology device use (eg, cell phone, iPad) or nightmares at least once per week. Additionally, survey participants reported a mean sleep duration of 6.1 hours per day, which is less than the recommended 7 hours of sleep for US adults [26]. Our study demonstrated that health care workers at highest risk of insomnia included those who were not physicians, were not male, were Hispanic, and directly cared for patients with COVID-19. We also demonstrate that sleep disturbances in health care workers during the pandemic are related not only to work demands and personal health but also to children and family as well as to the pandemic itself, including worry about the response to the COVID-19 pandemic. Given the strong association between sleep and burnout among health professionals, it is not surprising that a high number of the surveyed health care workers reported burnout [5].

The results of this study are consistent with prior studies demonstrating a high prevalence of insomnia in nurses and physicians caring for patients with COVID-19 on the front lines [8]. In a systematic review and meta-analysis, Salari et al [8] reported on the increase of insomnia in frontline nurses near 35% and in frontline physicians near 42%, however they did not report on a subgroup analysis of insomnia in women. In the meta-analysis by Pappa et al [7], the prevalence of insomnia in health care workers during the COVID-19 pandemic was near 40%, and the authors did not specifically report the prevalence of insomnia in women frontline health care workers. Our data are also in accord with those in a study by Trockel et al [5], who demonstrated that sleep-related impairment is an occupational hazard associated with increased medical-related errors and burnout. In comparing our sample to the general US physician population, we found higher rates of sleep disturbances and insomnia risk than in the general population of practicing physicians [12,27,28]. Our study findings of sleep disruption due to device use are consistent with findings demonstrating increased nightly screen time in health care workers and reports of doomscrolling [3,4].

Our findings have many clinical implications. Frontline health care workers involved in the direct care of patients with COVID-19 report decreased sleep time, increased insomnia, nightmares, fears for their safety, increased clinical workload, and concerns for their family, which reportedly impact their sleep. Health care workers are at high risk for the development of psychological distress and medical errors [5,29]. The professional environment for health care workers has drastically changed during the pandemic, which has brought challenges related to increased workload, reduced protective equipment and resources, rapidly evolving protocols, relocation of intensive care settings, fear of viral transmission, and social isolation from supportive networks [30,31]. Given this unfolding environment, the findings of sleep disturbances of frontline health care workers are not surprising. 

The study findings are particularly concerning given the natural course of insomnia and potential for long-term psychological impact, as seen in health care workers during prior severe acute respiratory syndrome (SARS) outbreaks [32-34]. In a recently reported longitudinal study on insomnia, nearly half (42%) of patients who had insomnia initially (as measured by the ISI) also had insomnia 5 years later, demonstrating a persistent course [35]. Likewise, studies on the mental health of health care workers who were active during the 2003 SARS epidemic suggest the psychological consequences may persist months to years after the epidemic ends [34]. While noting long-term impacts of mental health, it is also important to consider the implication of potential medical errors due to sleep loss by these health care workers [6]. Studies of sleep disorders in health care workers during prior infectious outbreaks are in concert with our study findings, and they forecast the potential longitudinal psychological impact on the sleep health of our frontline health care colleagues.

### Limitations

Although social media platforms are widely used, a limitation of this study may be that a sample recruited through social media platforms may not be representative of health care workers in the US and around the world. To combat time period bias, we asked participants to complete the survey while thinking of the month in which they were most clinically active in caring for patients with COVID-19. Although this approach may lead to biased results, we felt it would provide results that were most representative of the respondents’ experiences while they were serving on the front lines during the COVID-19 pandemic. Due to the nature of a worldwide pandemic, the incidence of COVID-19 is distributed differently across geographic areas; therefore, the time frame of reflection may differ between respondents. Because the focus of our survey was on sleep, it is possible that health care workers who were more likely to experience sleep disturbances completed the survey. Given the prevalence of sleep disorders in the general US population of 8%-19% [36,37], our sample does not appear to overrepresent people with sleep disorders. Our sample did not contain a sufficient number of nonphysician health care workers to characterize the sleep quality of nonphysician workers (eg, physician assistants, nurse practitioners, nurses, pharmacists, allied health workers) during the COVID-19 pandemic. We also asked the participants to complete the survey questions by reflecting on a time when they experienced the greatest clinical intensity and risk of COVID-19 transmission; this may have led to inconsistencies in our data. All data were self-reported, and we do not have objective measures of sleep duration and quality in this sample. We also do not have longitudinal data on our sample, limiting our ability to draw longitudinal conclusions. Furthermore, when using data obtained from social media, it is difficult to capture the accurate outreach and link clicks; for example, if an individual shares the link but not our original post, we cannot calculate the outreach and metrics. Therefore, the actual metrics may be higher than we were capable of measuring.

Prior to the pandemic, sleep and burnout issues existed among health care workers; however, strategies to improve and implement change are lagging behind. The reporting of these issues by frontline health care workers caring for patients with COVID-19 during the pandemic is not surprising. Studies demonstrating successful interventions to impact sleep and burnout are increasing, and they recommend interventions focused both at the individual and the organizational level. Examples of these recommendations include reducing work hours while maintaining the same salary, allowing schedule flexibility, behavioral wellness, and curricular interventions [38-41].

### Conclusion 

During the COVID-19 pandemic, almost all the health care workers we surveyed on social media reported poor sleep, with nearly half of respondents reporting moderate to severe insomnia and over half reporting burnout. Immediate interventions to improve the sleep and well-being of health care workers, discourage nocturnal social media use, and discourage doomscrolling are needed to strengthen the ability of these workers to continuously meet the daily demands of the COVID-19 pandemic. Concerns regarding clinician well-being are not new. Moreover, health systems and national organizations are placing greater emphasis on systemic change [38]. Practical frameworks for creating wellness exist; however, senior-level champions are critical for implementation [42]. Considering that sleep is a modifiable factor of our physical and mental health, prioritizing it may help mitigate the health risks associated with the pandemic and its potential longitudinal impacts on sleep and health.

## Abbreviations

ISI: Insomnia Severity Index
OR: odds ratio
PSQI: Pittsburgh Sleep Quality Index
R.E.S.T.: Recommending Essential Sleep Time
REDCap: Research Electronic Data Capture
